# Elementary Flux Mode Analysis Revealed Cyclization Pathway as a Powerful Way for NADPH Regeneration of Central Carbon Metabolism

**DOI:** 10.1371/journal.pone.0129837

**Published:** 2015-06-18

**Authors:** Bin Rui, Yin Yi, Tie Shen, Meijuan Zheng, Wenwei Zhou, Honglin Du, Yadong Fan, Yongkang Wang, Zhengdong Zhang, Shengsheng Xu, Zhijie Liu, Han Wen, Xiaoyao Xie

**Affiliations:** 1 School of Life Science, Anhui Agricultural University, Hefei, China; 2 Key Laboratory of Information and Computing Science, Guizhou Province, Guizhou Normal University, Guiyang, China; 3 Key Laboratory of Plant Physiology and Development Regulation, Guizhou Province, Guizhou Normal University, Guiyang, China; 4 Guizhou Academy of Testing and Analysis, Guiyang, China; Universidad de La Laguna, SPAIN

## Abstract

NADPH regeneration capacity is attracting growing research attention due to its important role in resisting oxidative stress. Besides, NADPH availability has been regarded as a limiting factor in production of industrially valuable compounds. The central carbon metabolism carries the carbon skeleton flux supporting the operation of NADPH-regenerating enzyme and offers flexibility in coping with NADPH demand for varied intracellular environment. To acquire an insightful understanding of its NADPH regeneration capacity, the elementary mode method was employed to compute all elementary flux modes (EFMs) of a network representative of central carbon metabolism. Based on the metabolic flux distributions of these modes, a cluster analysis of EFMs with high NADPH regeneration rate was conducted using the self-organizing map clustering algorithm. The clustering results were used to study the relationship between the flux of total NADPH regeneration and the flux in each NADPH producing enzyme. The results identified several reaction combinations supporting high NADPH regeneration, which are proven to be feasible in cells via thermodynamic analysis and coincident with a great deal of previous experimental report. Meanwhile, the reaction combinations showed some common characteristics: there were one or two decarboxylation oxidation reactions in the combinations that produced NADPH and the combination constitution included certain gluconeogenesis pathways. These findings suggested cyclization pathways as a powerful way for NADPH regeneration capacity of bacterial central carbon metabolism.

## Introduction

Aerobic life is constantly suffering from the hazard attack of oxidative stress [1]. When organisms use O_2_ to ensure that ATP production is enhanced via the process of oxidative phosphorylation, they are exposed to reactive oxygen species (ROS), such as superoxide (O_2_^–^), hydrogen peroxide (H_2_O_2_), and hydroxyl radical (OH•) [2,3]. The central carbon metabolic system plays a crucial role in oxidative stress responses [4,5]. Certain metabolites within the system and their derivatives possess the capacity to anti-oxidize and repair oxidative damage. Packer et al. found that a-lipoic acid and its derivatives have antioxidant activity [6]. TBrookes et al. and Fedotcheva et al revealed that a-ketoglutarate is able to eliminate ROS non-enzymatically and generate succinate, thereby contributing to the elimination of mitochondrial oxidative stress [7,8]. The research of Wu JL et al showed that adding of L-malate to the culture medium can relieve oxidative stress, and enhance the anti-oxidation system[9].

In these anti-oxidative metabolites, it is reduced nicotinamide adenine dinucleotide phosphate (NADPH) that has the central role in the cells' anti-oxidative defense strategies in most organisms. It is the shared carrier for all the cellular reducing power and the primary participant in anti-oxidation[10]. Through breaking the C-C bond and releasing chemical energy, a number of enzymes in the central carbon metabolism system enable NADPH regeneration from NADP^+^ [10]. Therefore, the NADPH regeneration capacity of the central carbon metabolism system has been increasingly investigated [4,5]. For example, Singh et al. found that several different sub-networks would be formed in the central carbon metabolism system under oxidative stress in *Pseudomonas fluorescens*, which results in more NADPH generation and an inhibition of NADH generation[11]. In *Escherichia coli*, we also found that oxidative stress could induce a transition in the central carbon metabolism system from its normal condition to a sub-optimal condition, where more NADPH could be generated[12].

Besides, NADPH provides basic reducing equivalence for reductive whole-cell biotransformation and NADPH-dependent enzymes play important roles in manufacturing industrially valuable compounds. As a result, the bioavailability of NADPH within the cell is often the rate-limiting factor for the over-production of desired compounds and improving NADPH regeneration capacity becomes one of the objects of current metabolic engineering[13]. Siedler et al. and Chin et al. attempted to increase the regeneration of NADPH by deleting pfkA and pgi in *Escherichia coli*[14]. Martı´nez et al. had achieved this goal by replacing *Escherichia coli* NAD-dependent glyceraldehyde 3-phosphate dehydrogenase (GAPDH) with a NADP-dependent enzyme from *Clostridium acetobutylicum* [15]. Jiang et al. had expressed NADP-dependent enzyme from *Clostridium acetobutylicum* in *Corynebacterium glutamicum* and almost trebled the NADPH availability [16].

Although the central carbon metabolism system is not very complicated, combinations of its various sub-pathways result in substantial flexibility[17] and diversified physiologically feasible conditions[18], which ensure abundant ways to regenerate NADPH. In addition, the specific flux distribution within central carbon metabolism determined which NADPH-generating enzyme and which pathway were utilized to fulfill the physiological demand. Therefore, systematic theoretical analysis of the system is necessary to elucidate possible ways to increase NADPH availability effectively. This will not only identify more potential targets to resist oxidative stress under physiological conditions, it will also shed light on ways to use the anti-oxidation ability of bacteria themselves during anti-oxidation treatments. Furthermore, together with chemical biosynthesis, it could enable us to combine or decouple different pathways to fulfill the demand in NADPH regeneration.

The theoretical analysis presented in this work uses the concept of “elementary flux modes/extreme pathways” (EFMs) as its base [19,20,21]. An elementary mode can be defined physiologically as the smallest set of enzymes that enable a mode to persist in a certain fixed direction with a stable metabolic flux distribution[22]. Based on its mathematical definition, an elementary mode is the generating basis for the metabolism system’s solution space. Elementary modes cannot be represented linearly by other vectors in the solution space[20]. Any flux distribution in the living cell could be decomposed into a linear combination of EFMs. Thus, once all elementary modes of the metabolism system are obtained, the shape and characters of the systems’ solution space can be fully grasped.

Elementary flux modes have been applied successfully in metabolic network analysis for several cases. For example, Figueiredo et al. established all potential NAD synthesis and degradation pathways by use of EFMs, and found that substrate specificity differed significantly in species like yeasts and humans[23]. Through computing and screening the EFMs of yeast metabolic systems based on thermodynamic restriction, Jol et al. obtained thermodynamically-available EFMs and therefore narrowed the solution space for the yeast metabolism system, which contributed to a deeper understanding of yeast metabolism[24].

In this study, using a central carbon metabolism networks modified from that one reconstructed by Palsson et al.[25], all EFMs were computed by utilizing the elementary mode method. Then based on the flux of NADPH regenerating enzymes, the clustering analysis was conducted on the EFMs with high NADPH productivity with a self-organizing map (SOM) clustering algorithm. The clustering results were used to investigate the flexibility and mutual relationships between each NADPH regenerating enzyme. We derived the relationship between total NADPH yield and the flux of each NADPH regenerating enzyme. In addition, it was found that various reactions could be combined to constitute different open cyclization pathways that operated in a high abundance, which are necessary to achieve high NADPH generation. Such cyclization pathways usually contain one or two step of decarboxylation oxidation reactions that generate NADPH and certain gluconeogenesis pathways.

## Theory and Methods

### Metabolic networks and data sources

The metabolic networks for EFM computing and subsequent analyses are detailed in S1 Appendix. Specifically, the total network includes the following pathways: Embden-Meyerhof-Parnass pathway, the pentose phosphate pathway, the tricarboxylic acid cycle, the anaplerotic pathway, the Entner-Doudoroff (ED) pathway, the central nitrogen metabolism pathway, exchange reactions and biomass synthesis. The *E*.*coli*’s biomass composition was taken to represent the biomass synthesis. In total, 72 reactants and 97 reactions are involved, of which 20 reactions are responsible for exchange of intracellular and extracellular materials. The network captures a full representation of the central carbon metabolism of bacterium In contrast, this network does not contain the compartment information. However, it would also be applicable to eukaryotic organisms, since the transportations between compartments would not affect the elementary flux mode enumeration. The reversibility of the reactions is defined mainly based on original articles and the BRENDA database[26]. For example, in terms of malic enzymes, two forms (i.e. NAD^+^ dependent and NADP^+^ dependent) were considered in this study. No other restriction was applied in the computation.

### Definition of NADPH fluxes

The NADPH fluxes are as follows: “G6PDH2r” catalyzes G-6-PDH to simultaneously generate 6-phospho-D-glucono-1,5-lactone and NADPH. “GLUDy” catalyzes Glutamate to simultaneously generate 2-Oxoglutarate and NADPH. “GLUSy” catalyzes the transamination between 2-Oxoglutarate and Glutamine, thereby generating 2-molecular Glutamate by consuming 1 NADPH molecule. “GND” catalyzes 6-Phospho-D-gluconate to release carbon dioxide (CO_2_) and to simultaneously generate D–Ribulose-5–phosphate and NADPH. “ICDHyr” catalyzes isocitrate oxidation to generate 2-Oxoglutarate and NADPH. “ME2” produces PYR and NADPH via malate oxidative decarboxylation. Thus, NADPH regeneration flux of the central carbon metabolism system could be defined as:

$${\text{V}}_{\text{NADPH}}={\text{V}}_{\text{G}6\text{PDH}2\text{r}}+{\text{V}}_{\text{GLUDy}}-{\text{V}}_{\text{GLUSy}}+\;{\text{V}}_{\text{GND}}+\;{\text{V}}_{\text{ICDHyr}}+\;{\text{V}}_{\text{ME}2}$$ (1)

### EFM analysis

When computing the EFM it can be considered as an extreme ray enumeration of polyhedral cones, which can be conducted with many software programs, such as Metatool, YANA, and EFM tool, etc.[27,28]. This study used the Metatool, which is a software package compatible with Matlab, from Schuster group. Most previous oxidative stress or NADPH producing experiments have been performed in glucose minimal medium[4,5,11]. In order to match these experiments, the glucose exchange reaction was set to be input-only and irreversible, other substrates that represented carbon sources were set to be output-only and irreversible, and the remaining materials were set to be able to be bilaterally exchanged.

### Cluster analysis

#### Clustering parameters

The *selforgmap* cluster package integrated in Matlab was adopted to perform SOM clustering of input samples. The parameter settings were as follows: number of training steps = 200 for initial coverage of the input space; initial neighborhood size = 2; and maximum number of training = 500. A two-dimensional SOM network was used and its structural function was set as hexagonal.

#### Clustering inputs

In the top 10% of samples with maximal NADPH regeneration rate, clustering was performed for the reactions related to carbon atom skeleton changes. In total, a subset of 45 reactions was selected from the total of 97, including several reactions of nitrogen metabolism pathways. The flux values of these reactions were used as the input for the clustering.

#### Clustering number

The use of SOM clustering can avoid the defect of initial value sensitivity in K-means clustering[29]; however, its disadvantage is that the optimal clustering number cannot be determined automatically. In this study, the 2-dimensional scales (n1 and n2) of the SOM network were set to vary from 2 to 8, so a total of 49 potential cluster networks could be obtained, for each cluster analysis that was performed.

One criterion for evaluation of clustering quality is to derive an intra-group sum of squares of deviations that is as small as possible, with a maximal inter-group sum of squares[30]. Then, the quality of clustering analysis can be reflected through the comparison of the two sums of squares. The Hotelling-Lawley Trace value of the multivariate variance test is a good option due to the multiple attributes of the clustering. Reaction stoichiometric constraints result in interdependent fluxes of correlated reactions, thus the determinant value of the covariance matrix was zero during trace value computation. Consequently, the inverse matrix of the intra-group sum of squares does not exist, so the trace value could not be calculated. However, it is worth noting that the emphasis of this study is the distribution of every reaction, so the initial objective could not be fulfilled if some observations were eliminated or changed accordingly through dimensionality reduction. For this reason, the statistical quantity of a Levene test in single-variance homogeneity analysis was used for reference, where the square of error between a single point and the mean value was replaced by the square of Euclidean distance between the flux distribution and the mean value. Then, the value Q was derived as an index for clustering quality:

$$Q=\frac{(N-k)*\sum_{i=1}^{k}N_{i}*D{\left(V_{i}-V\right)}^{2}}{(k-1)\sum_{i=1}^{k}\sum_{j=1}^{N_{i}}D{\left(V_{ij}-V\right)}^{2}}$$ (2)

where N represent the total number of flux modes, *k* is the number of clusters; N_*i*_ is the number of samples within the *i*th cluster; D is the Euclidean distance; and V, V_*i*_, and V_*ij*_ represent the flux value of the mean of the total sample, mean of the *i*th cluster, and the *j*th sample of the *i*th cluter, respectively.

Another index used to demonstrate clustering quality was the degree of balance between different clustering results. Since the number of samples was constant, the standard deviation (SD) of sample number for each category could be considered as an indicator of the degree of balance in clustering. Among the 49 clustering results, the optimal cluster number was determined by combining Q and SD.

### Thermodynamic analysis of elementary mode

The most common criterion to evaluate the thermodynamic availability of a reaction is the change in Gibbs free energy under physiological condition. The Gibbs free energy for a given reaction (or reaction group) can be calculated as [31,32]:

$$\Delta_{r}G'=-\sum_{i=1}^{m}n_{i}\Delta_{f}G_{i}{'}^{{}^{\circ}}+RT\;\text{ln}(\prod_{i=1}^{m}X_{i}^{n_{i}})$$ (3)

where *Δ*_*f*_ *G*_*i*_ *'°* is the formation free energy of chemical compound *i*, *m* is the number of compound categories that participate in the reaction, *n*_*i*_ is the stoichiometric coefficient of compound *i* in the reaction, *R* is the universal gas constant, *T* is Kelvin temperature of 37°C that is optimal for growth of most organisms, and *x*_*i*_ is the activity or concentration of compound *i*.

The concentration value of corresponding metabolites in *E*. *coli* from Chassagnole et al. and Bennett et al. was used as the representative value under normal physiological conditions [33,34]. Since the concentration of metabolites varied under different conditions[35], the range in concentrations of intracellular metabolites was set to a minimum of 10^-5^M and a maximum of 0.02M, and the intracellular H ion concentration was set to pH = 7 as previously described[32]. As Δ_f_G_i_'° could not be measured for most materials, the group contribution method was used to calculate the value [36,37].

The glucose PTS transfer and reaction system was involved in computing the free energy of some reaction combinations. Since the extracellular glucose concentration far outweighed the intracellular concentration, and the net charge of glucose molecules was zero, it was therefore not subject to a transmembrane potential difference[32]. Thus, the free energy of the PTS transmembrane movement was set to zero during the computation. For ME-TCA cycle and ME-GLX cycle, the ubiquinone is diffusing into the membrane and its chemical potential is complicated by this process, which prevent a precise calculation of Δ_f_G' value from Eq (3). Thus, we only calculate the physiological Δ_f_G' of the two cycles using the values of the constituent reactions from literature [36,37].

## Results

### Computation of EFMs

The reconstructed metabolic network consisted of 72 metabolites and 97 reactions, 20 of which involved material exchange between the cellular system and the external environment. Considering glucose as the only carbon source resulted in 234,472 elementary modes, of which 219,742 had NADPH regeneration fluxes above zero—this indicated that the solution space in the dimension of NADPH regeneration flux was not distributed symmetrically around the zero point.

### Capacity analysis of the NADPH generating enzyme

Due to the fact that the clustering objects were edges of a continuous convex polyhedron, an SOM clustering algorithm method was employed. SOM is not just a clustering algorithm, but also a dimension-reduction algorithm. It can project high-dimensional data to a nodal plane, thereby achieving visualization of the data, which made it suitable for this study[29]. SOM can also avoid sensitivity to the initial cluster center of K-Mean clustering.

Since our main interest was the NADPH regeneration capacity of the system that involves the transfer of carbon skeletons, the clustering observation items were 45 primary skeleton reactions. This study aimed to assess the strength of NADPH regeneration capacity, and how the metabolism system adjusts to provide maximum NADPH generation. Therefore, 20,000 elementary modes with maximal NADPH total generation fluxes were selected as samples for clustering.

**Fig 1:**
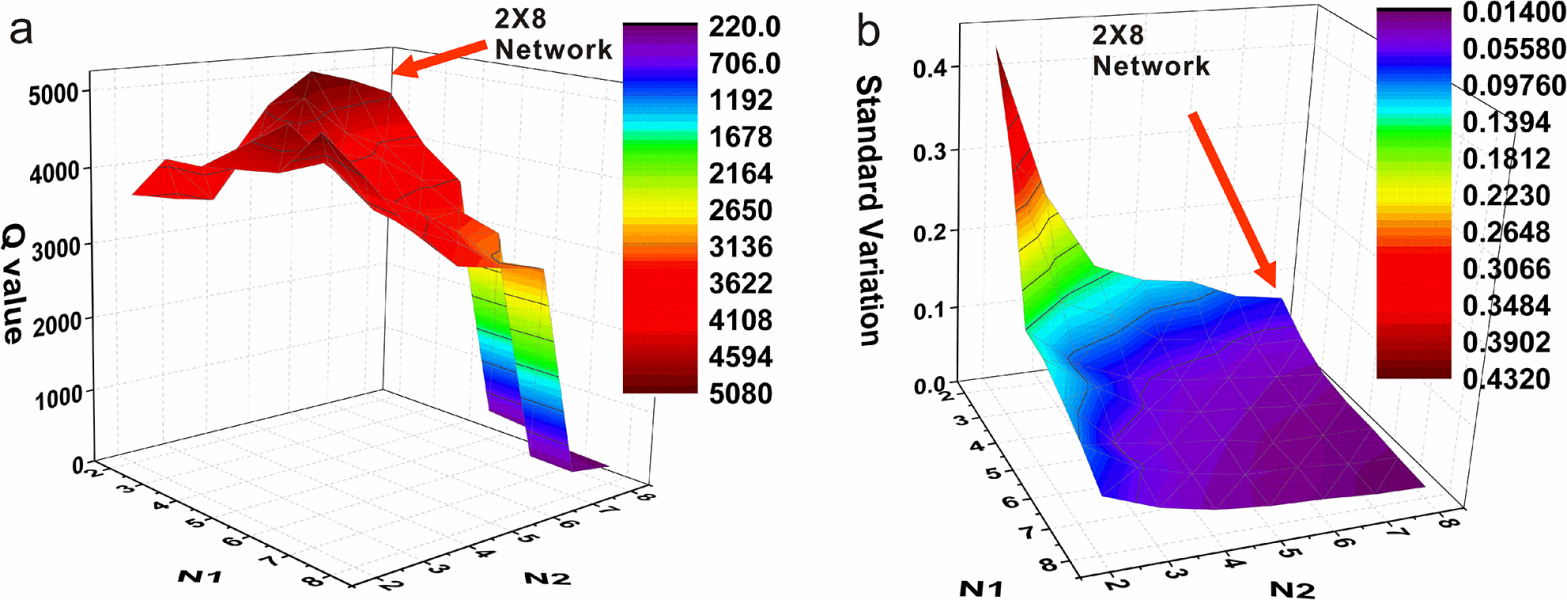
Q value and dispersion of different SOM clustering results. Fig 1a shows Q values for different SOM clustering results, and Fig. 1b shows the standard deviation of different SOM clustering results. Coordinate X is the first dimension N1 in SOM clustering and coordinate Y is the second dimension N2, so N1*N2 is the number of clusters for each cluster. Coordinate Z in Fig 1a and b is the Q value and standard deviation of the cluster, respectively. For 3X5, 5X3, 2X7, 2X8, and 8X2, the Q value is relatively high. Among the clusters with large Q values, those with small flux standard deviation (i.e. sample dispersion) were selected (Fig 1b).

To determine the optimal number of clusters and network shape, the two dimensions of 2D SOM were changed so that 49 different clustering results were derived. The Q value (ratio of the sum of squares between group to the sum of squares within group) was taken as the primary testing result, and the response surface chart is shown in Fig 1A. It is clear that networks 3X5, 5X3, 2X7, 2X8, and 8X2 have relatively high Q values, so they were good clustering schemes. Furthermore, among the clustering results with large Q values, clusters with small flux standard deviations (i.e. sample dispersion; Fig 1B) were considered as preferential cluster schemes. Ultimately, the 2X8 SOM network was selected for clustering.

**Fig 2:**
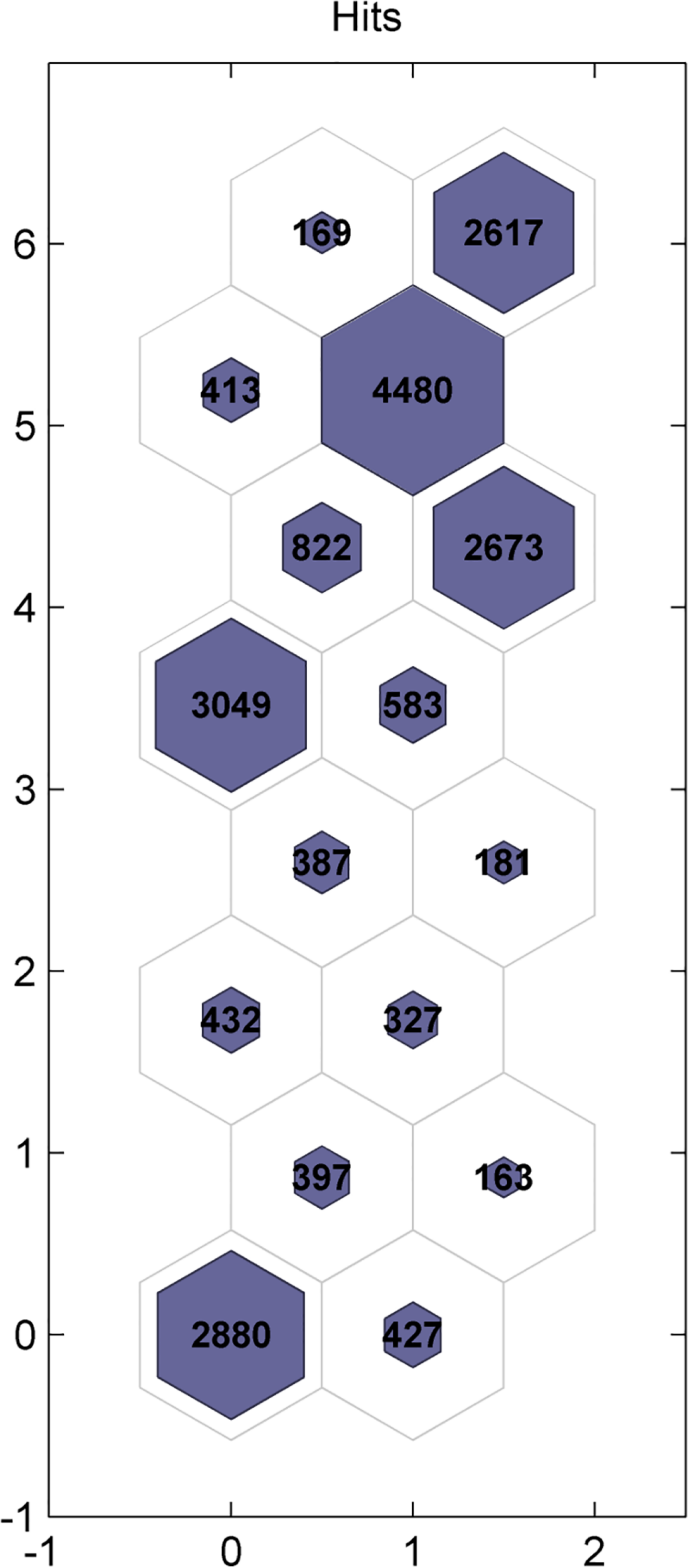
Optimal clustering results. The horizontal coordinates vary from 0 to 2, and the longitudinal coordinates range from 0 to 8, so the number of clusters is 16. Each hexagon represents one category, and the figure in the hexagon is the number of elementary modes.

The optimal clustering results are shown in Fig 2. The largest cluster had over 5,000 samples, while some smaller clusters had less than 1000 samples. These clustering results are a natural reflection of sample characters—because the elementary modes are edges of super pyramids[19]; their spatial distribution depicts a large quantity near the center of the sample space, and a small quantity near the pyramid's apex.

**Fig 3:**
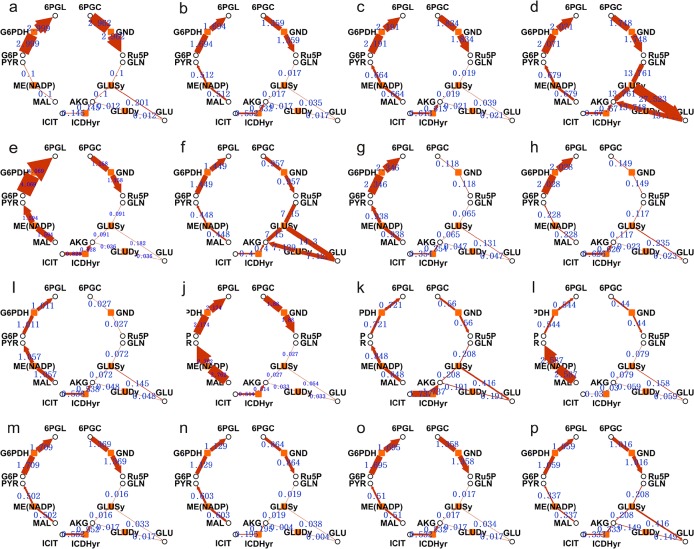
Flux of NADPH generating enzymes of the cluster center of 16 categories. The yellow solid blocks represent the enzymes, the black circles are the metabolites, and the blue number is the flux value of cluster center of each category. The width of the brown edges is proportional to the passed flux. The abbreviations of enzymes and metabolites are as shown in S1 Appendix and S2 Appendix.

Mean flux values of the cluster center are shown in Fig 3. As clustering was performed for only NADPH-related reactions, only six NADPH-related reactions are listed. The value of each sub-graph is the average flux of each cluster center. In Fig 3A, G6PDH and total NADPH fluxes are large, whereas GND flux is relatively small, suggesting that partial materials were shunted by the EDD pathway. All six reactions in Fig 3B show small fluxes. In Fig 3C, the G6PDH and ME fluxes are relatively large, while the fluxes of other pathways are small. In Fig 3D, the fluxes of GLUDy and GLUSy generally increase, while the other fluxes are small. In Fig 3E, the ME pathway dominates. In Fig 3F, G6PDH and GND reach the peak value at the same time, which results in a large total NAPDH flux.

Briefly, either G6DPH or GND could bear a large flux themselves, so they are major contributors to total NADPH generation. Specifically, the maximal total NADPH flux can be obtained if both G6PDH and GND fluxes are high while other reactions have nearly zero flux. Meanwhile, ME plays an important role in total metabolism flux. One certain suboptimal value of total NADPH flux is achieved by increasing ME flux and decreasing other fluxes. Singh et al. reported that serial operation of *E*. *coli*’s metabolic enzymes could form an NADPH generation network, during which ME flux demonstrated significant up-regulation. Moreover, due to the poor correlation between ME and G6PDH, the fluxes of both reactions could be increased at the same time, through certain biological operations.

Contrarily, ICDH contributes less to NADPH flux, which might be attributed to the small ICDH flux. Only when the whole tricarboxylic acid cycle is at a high level does the ICDH contribution to NADPH increase to 50%. That indicates that although ICDH is an important NADPH-generating enzyme during general physiological functioning, it does not have the potential to increase. In addition, GLUDy and GLUSy always have similar fluxes, so the generated and consumed NADPH cancels each other out. Therefore, this branch does not make an obvious contribution to NADPH generation.

Based on the above distributions, it could be observed that it is difficult to amplify the fluxes of these reactions simultaneously without changing the topology structure of the metabolism network, because of the trade-off between different reactions. To activate the NADPH generation capacity of the central carbon metabolism pathway, emphasis should be put on G6PDH, GND, and ME rather than on the other two pathways that have little space for improvement.

### Discovery of cyclization pathway with high NADPH regeneration rate and their structure analysis

**Fig 4:**
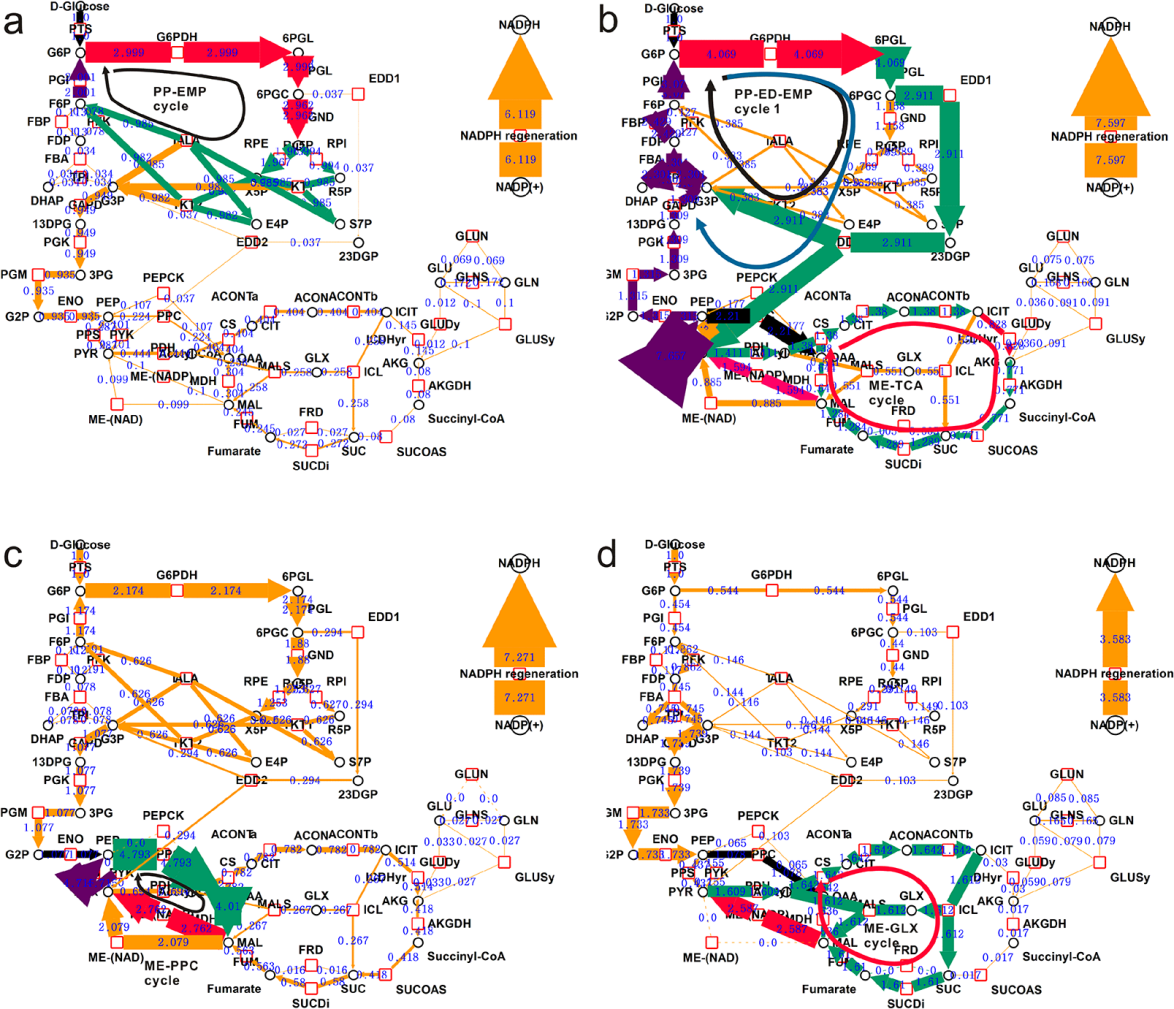
Six cyclization pathways supporting high NADPH generation. Red hollow blocks represent the enzymes, the black circles are the metabolites, and the blue number is the flux value of cluster center of each category. The width of the brown edges is proportional to the passed flux. The abbreviations of enzymes and metabolites are as shown in S1 Appendix and S2 Appendix. The reactions in black color provide the initial carbon skeleton for a cyclization pathway. The reactions in red color are part carrying out oxidative decarboxylation and NADPH generation. The purple pathway is the gluconeogenesis pathway, and the yellow arrow represents the reaction not in the target cycle. In Fig 4a, the cluster is the *a*th cluster in Fig 3, containing the PP-EMP cycle. In Fig 4b, the cluster is the *e*th cluster in Fig 3, containing PP-ED-EMP cycle 1, PP-ED-EMP cycle 2 and ME-TCA cycle. In Fig 4c, the cluster is the *j*th cluster in Fig 3, containing ME-PPC cycle. In Fig. 4d, the cluster is the *j*th cluster in Fig 3, containing ME-GLX cycle.

Several different cyclized flux modes with high NADPH availability were observed in the clustering results, namely the PP-EMP cycle, PP-ED-EMP cycle 1, PP-ED-EMP cycle 2, and ME-PPC cycle, ME-GLX cycle and ME-TCA cycle (Table 1 and Fig 4). The chemical reactions of each of the six modes were added according to the individual occurrence coefficient of each reaction, to derive the net reaction equation for each reaction (Table 1). The chemical reaction equation shows that the PP-EMP cycle, ME-TCA cycle and ME-GLX cycle break the C-C bond and releases energy, thereby generating NADPH, while the other three modes generate NADPH by transferring reductive equivalent from NADH.

**Table 1:** Composition of the four flux combinations that generate NADPH.

Enzyme cycle	Enzyme composition	Reactant	Product
PP-EMP cycle	PTS^a^+G6PDH^b^+PGL+GND+RPE+RPI+TKT1+TALA+TKT2+PGI	D-Glucose^c^ + PEP + 2 NADP^+^+ H_2_O + R5P + X5P	PYR + 2 NADPH + 2 H^+^ + CO_2_ + G6P + F6P + GAP
PP-ED-EMP Cycle 1	PTS+G6PDH+PGL+EDD1+EDD2+PPS-ENO-PGM-PGK-GAPD-TPI-FBA-FBP-PGI	D-Glucose + NADP^+^ + 2H_2_O + PEP + 2 ADP + 2 3PG + NADH + 2 DHAP	G6P + PYR + NADPH + 2 GAP + AMP + 2 Pi + 2PG + ATP + NAD^+^
PP-ED-EMP Cycle 2	PTS+G6PDH+PGL+EDD1+EDD2-FBA-FBP-PGI	D-Glucose + PEP + NADP^+^ +DHAP + ADP	ATP + G6P + 2 PYR + NADPH + H+
ME-TCA cycle	PDH+CS+ACONTa+ACONTb+ICDHyr+AKGDH+SUCOAS+SUCDi+FUM+ME+PPC	2 NAD^+^+ 2 NADP^+^+ 3 H_2_O +ADP + Ube-8+ PEP	3 CO_2_ + 2 NADH + 2 NADPH + 2 H^+^+ ATP + Ubl-8
ME-PPC cycle	ENO+PPS-MDH+ME+PPC	2PG + ADP + NADH +NADP^+^	PEP + AMP + Pi + NADPH + NAD^+^
ME-GLX cycle	PDH+CS+ACONTa+ACONTb+ICL+MALS+SUCDi+FUM+ME+PPC	NAD^+^+AcCOA+Ube-8+ 2H_2_O+NADP^+^+PEP	CO_2_+NADPH+3H +COA+MAL+Ubl-8+NADPH+Pi

a Enzyme abbreviations are shown in S1 Appendix.

b Indicates constitution of certain mode via forward reaction (+) or backward reaction (-).

c Metabolite abbreviations are shown in S2 Appendix.

Afterwards, the structural constitutions of the elementary modes with high NADPH generation capacity were analyzed. The mode corresponding to Fig 4A is the PP-EMP cycle. NADPH generation is accelerated by increasing G6PDH and GND fluxes, which is, apparently, the most direct way to increase *E*. *coli*’s NADPH generation. As seen in Table 1, this cycle consists of 10 reactions that involve activation of some gluconeogenesis reactions. In this mode, F6P is regenerated from pentaglucose through the non-oxidative branch of the pentose phosphate pathway, which is then transferred into G6P through the process of gluconeogenesis, participates in the pentose phosphate pathway again, and finally generates NADPH. This cyclization broke down one mole C-C bond and rearranged the C-C skeleton of the carbohydrates with a yield of 2 mole NADPH.

The two NADPH generation modes in top of Fig 4B are the PP-ED-EMP cycle 1 and PP-ED-EMP cycle 2, which have some common characteristics: total NADPH flux is achieved by increasing the G6PDH flux (4.069) and ED flux (2.911) pathways, whereas the flux of the GND pathway is almost zero, and the flux of the non-oxidative branch of the pentose phosphate pathway is also very small. The metabolic flux generates G3P and PEP through the ED pathway, from which the two modes become different. As the slightly bigger cycle, the PP-ED-EMP cycle 1 is composed of 14 reactions (Table 1). In this mode, PEP generated from the ED pathway produces G3P through the gluconeogenesis pathway, which transfers back to G6P through the same pathway. Then G6P participates in the G6PDH pathway. Thus, a relatively complete gluconeogenesis pathway is utilized in this mode. On the other hand, the PP-ED-EMP cycle 2 is relatively smaller, consisting of 8 reactions. G3P is generated through the ED pathway, and then through the upper half of the gluconeogenesis pathway, G6P is generated that could be re-used in the G6PDH pathway. The cycles rely on the ED pathway and gluconeogenesis transfer to perform G6PDH decarboxylation of carbon metabolism flux, and to ultimately generate NADPH.

The other three modes are related to malic enzyme. In bottom of Fig 4B, besides the already-known PP-ED-EMP cycle, the ME-TCA cycle could also be found, where ME reaches a value as 1.594. In this mode, PDH, CS, ACONTa, ACONTb, ICDHyr, AKGDH, SUCOAS, SUCDi, FUM, ME and PPC constitute a loop similar to TCA cycle. The PPC step provide the fueling block for this cycle and Malic enzyme (NADP+) and PDH was used to circumvent the MDH step to obtain an extra NADPH production in addition to ICDH. The net result, slightly away from the TCA cycle, is to completely degrade the PEP molecule to 3 CO_2_ for 2 NADH and 2 NADPH regeneration.

In Fig 4C, the colored reactions represented ME-PPC cycle. In this mode, the ENO, PPS, MDH, ME, and PPC reactions constitute an open loop; PEP is first generated from 2PG, and OAA is then generated, which could produce MAL through the MDH reduction reaction. Then, the ME enzyme enables decarboxylation of MAL and NADPH regeneration. Meanwhile, PYR is generated, which produces PEP through the PPS reaction.

In Fig 4D, the ME-GLX cycle could be found where ME reach a value of 2.587 in contrast to a total NADPH flux of 3.58, suggesting that the metabolism system generate NADPH primarily based on ME under this condition. This mode was composed of 10 reactions similar to ME-TCA cycle. Contrarily, this cycle utilizes glyoxylate pathway to get across the carbon-losing step. The net effect of this cycle, unlike the ME-TCA cycle, is to combine the PEP and ACCOA into MAL with breaking one C-C bond of PEP and releasing one CO_2_. This process generates 1 mol NADH and NADPH.

### Common characters of NADPH regenerating modes

The above six modes have some shared characteristics. Firstly, to enable high NADPH productivity, all six modes form an open loop based on a combination of various metabolism pathways. The loop is necessarily open otherwise it is not allowed in thermodynamics. Secondly, these open loops are conservative in structure. There is always a carbon skeleton input reaction for each loop as shown in black in Fig 4. For PP-ED-EMP cycles and the PP-EMP cycle, the input reaction is the PTS pathway, while it is the ENO reaction for the ME-PPC cycle and PPC reaction for ME-GLX and ME-TCA cycles. Thirdly, they all have one or two decarboxylation oxidation reactions (represented as green arrows) to generate NADPH, namely, G6PDH, GND, ME and ICDH. In these loops, the different parts of the gluconeogenesis pathway are indicated as purple arrows in the thumbnail figure.

### Thermodynamic feasibility of the elementary flux modes

Chemical thermodynamic analysis is required to verify whether the elementary modes obtained from mathematical analysis can operate under normal physiological conditions. For chemical reactions/combinations, the framework for thermodynamic analysis is already relatively mature [31,32,36], but the activity or concentration value of each chemical compound is lacking.

To be representative, the values measured in the steady logarithmic growth phase were considered to be the stress-free under natural physiological conditions [33,34]. Based on the experience of Henry et al., the minimal and maximal activities of metabolites were set so as to compute the thermodynamic extreme value, at 0.01mM and 20mM, respectively[31]. The free energy of PTS transmembrane movement was set to zero during the computation.

**Fig 5:**
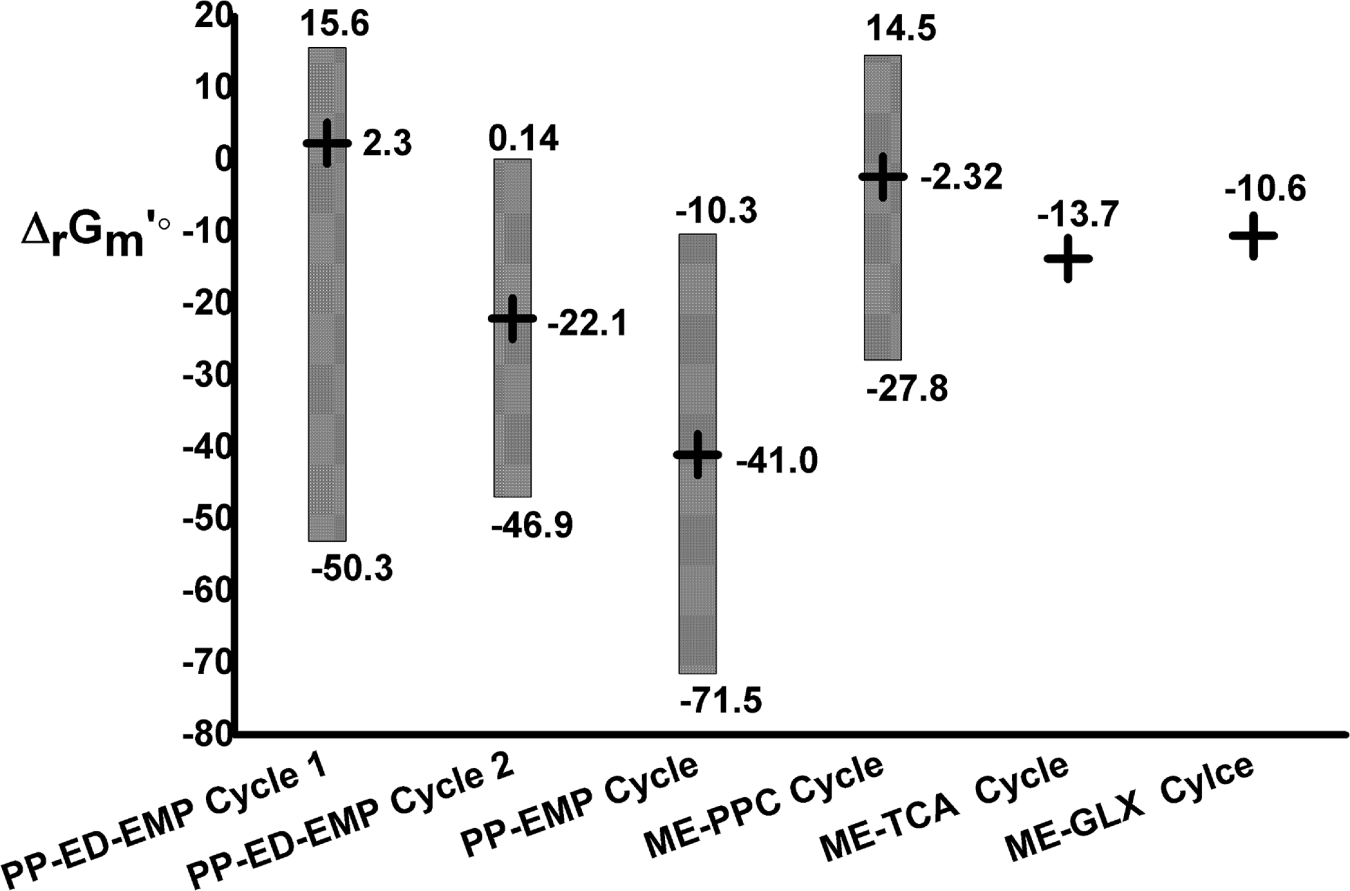
Gibbs free energy of four flux modes. The horizontal coordinates are mode categories, and the longitudinal coordinates are the Gibbs free energy obtained by the Group Contribution Method. The top end of the red bar chart is the highest possible Gibbs free energy, and the bottom end is the lowest possible Gibbs free energy, while the black crosses represent Gibbs free energy obtained from the concentration of intracellular metabolites without oxidative stress.

Then, the normal ⊿_r_G_m_’, highest possible ⊿_r_G_m_’, and lowest possible ⊿_r_G_m_’ were computed, with the results shown in Fig 5. The black cross symbol in the bar middle represents normal ⊿_r_G_m_’, the top end of the bar chart is the highest possible ⊿_r_G_m_’, and the bottom end is the lowest possible ⊿_r_G_m_’. For the PP-EMP cycle, the values were -40.9, -10.3, and -71.5 kCal/mol, respectively. For the PP-ED-EMP cycle 1, the values were 2.34, 15.6, and -53.0 kCal/mol. For the PP-ED-EMP cycle 2, they were -22.1, 0.144, and -46.9kCal/mol. For the ME-PPC cycle, they were -2.32, 14.5, and -27.8kCal/mol. The physiological⊿_r_G_m_’s of ME-GLX and ME-TCA cycle are both less than zero. From the thermodynamic values, it can be seen that the PP-EMP cycle, PP-ED-EMP cycle 2 and ME related cycles are physiologically preferred and can happen under natural physiological condition. The lowest possible ⊿_r_G_m_’ of the PP-ED-EMP cycle 1 was significantly less than zero, while the activity of metabolites changed while super-oxidative stress occurred[35]. This could probably induce the operation of the PP-ED-EMP cycle 1.

### Experimental observations of cyclized NADPH regenerating modes

The physiologically feasibility proposed by the thermodynamic analysis has placed our calculation and analysis on solidated and safe position. However, the prediction capacity and confidence of the framework should be confirmed in context of real experiments. After a thorough mining of current literature of related topics, most of these cyclized pathways calculated has indeed been reproduced in other experimental reports. Siedler et al. doubled the NADPH regeneration rate by deleting the phosphofructokinase gene pfkA in *Escherichia coli* and confirmed that it was due to a partial cyclization of the pentose phosphate pathway by ^13^C metabolic flux analysis [38]. Immediately afterwards, Siedler et al. had realized reductive whole-cell biotransformation with *Corynebacterium glutamicum*. TheΔ*pfkA* mutant strain reached a yield of 4.8 mol NADPH mol^-1^ glucose while theΔ*gapA* mutant reached a yield of 7.9 mol NADPH mol^-1^ glucose[39]. Both situations are resulted from the cyclization of pentose phosphate pathway, which is just about the PP-EMP cycle in this study. Additionally, when most organisms including *E*. *coli* are subject to oxidative stress, the PP-EMP cycle reduces the glycolysis pathway and increases the pentose phosphate pathway [12,40]. This is actually a combination of a stress-free metabolism network and the PP-EMP cycle.

Secondly, PP-ED-EMP cycles are also observed. Berger et al. investigated the flux distribution of *Pseudomonas* under oxidative stress from clinical infection, and found that carbon metabolism flux completely transferred from the EMP pathway to G6PDH. Moreover, most 6-phospho-D-glucono-1,5-lactone entered the ED pathway after G6PDH, rather than through the non-oxidative branch[41]. This situation is actually a combination of PP-ED-EMP cycles and the distribution of metabolic fluxes under normal conditions. Meanwhile, Lien et al. found by 13C-fluxomcis that *Pseudomonas fluorescens* SBW2 wild type underwent a significantly high flux in gluconeogenic pathway, which together with a vigorous ED pathway formed a PP-ED-EMP cycle 1 as in this study. This flux distribution had contributed to an obviously higher NADPH production than that of a mutant strain [42]. Moreover, Hanke et al. combined fluxomics and transcriptomics analysis to discover that *Gluconobacter oxydans* 621H degrade the glucose via partially cyclic Pentose Phosphate Pathway and Entner-Doudoroff pathway, which are no other than the EMP-PP cycle and PP-ED-EMP cycle 1[43].

Additionally, the ME related cycles are also common in physiological experiment. In a xylose-fermenting recombinant *Saccharomyces cerevisiae*, the ME-PPC cycle was activated by malic enzyme, malate dehydrogenase and pyruvate carboxylase. This metabolic shunt achieved a transhydrogenase-like shunt [44]. Dghim et al. investigated the capacity for cytosolic NADPH regeneration by NADP-dehydrogenases in the leaves of two hybrid poplar genotypes in response to ozone (O_3_) treatment. In one genotype, the increase in PPC activity was quite well correlated to the increase in NADP-ICDH activity and NADP-ME activity. This phenomenon resembled the activation of ME-TCA cycle [45].

## Conclusions

NADPH regeneration capacity of central carbon metabolism system was better understood through computation of its EFMs. An SOM clustering algorithm was applied to EFMs with high NADPH productivity, and 16 categories were obtained. The relationship between each NADPH generating enzyme and total NADPH flux was discussed. Several cyclic pathways that supported high NADPH generation were found, and proved to be totally feasible in cells via thermodynamic analysis. Comparison with previous studies suggested that these pathways were indeed NADPH generation strategies used by various organisms. Meanwhile, the pathways showed some shared characteristics: an open loop was formed based on the combination of various metabolism pathways, and one or two decarboxylation oxidation reactions in the loop generate NADPH; there are also certain gluconeogenesis pathways in some loop’s constitution. Our results suggested that analyzing the relationships between various metabolism pathways at an overall systematic level facilitates the understanding of metabolic-related changes—this not only indirectly validates the flexibility of the bacteria’s operation on metabolism branches, but provides a new perspective for designing combinations of different metabolic reactions to achieve desired cofactor availability and internal environment.

## Supporting Information

S1 Appendix Abbreviation. (DOC)

S2 Appendix Nomenclature. (DOC)

S1 Table The flux value of center of 16 clusters. (XLSX)

## References

[1] Apel K, Hirt H (2004) Reactive oxygen species: metabolism, oxidative stress, and signal transduction. Annu Rev Plant Biol 55: 373–399. 15377225 10.1146/annurev.arplant.55.031903.141701

[2] Cabiscol E, Tamarit J, Ros J (2000) Oxidative stress in bacteria and protein damage by reactive oxygen species. Int Microbiol 3: 3–8. 10963327

[3] Imlay JA (2013) The molecular mechanisms and physiological consequences of oxidative stress: lessons from a model bacterium. Nat Rev Microbiol 11: 443–454. 10.1038/nrmicro3032 23712352 PMC4018742

[4] Singh R, Mailloux RJ, Puiseux-Dao S, Appanna VD (2007) Oxidative stress evokes a metabolic adaptation that favors increased NADPH synthesis and decreased NADH production in *Pseudomonas fluorescens* . J Bacteriol 189: 6665–6675. 17573472 10.1128/JB.00555-07 PMC2045160

[5] Bin R, Tie S, Hong Z, Jiusheng C, Xiaosong P, Haiyan L, et al (2010) A systematic investigation of *Escherichia coli* central carbon metabolism in response to superoxide stress. BMC Syst Biol 4: 122–134. 10.1186/1752-0509-4-122 20809933 PMC2944137

[6] Li X, Liu Z, Luo C, Jia H, Sun L, Hou B, et al (2008) Lipoamide protects retinal pigment epithelial cells from oxidative stress and mitochondrial dysfunction. Free Radic Biol Med 44: 1465–1474. 10.1016/j.freeradbiomed.2008.01.004 18258206 PMC2597696

[7] Brookes PS, Freeman RS, Barone MC (2006) A shortcut to mitochondrial signaling and pathology: a commentary on "Nonenzymatic formation of succinate in mitochondria under oxidative stress". Free Radic Biol Med 41: 41–45. 16781451 10.1016/j.freeradbiomed.2006.03.019

[8] Fedotcheva NI, Sokolov AP, Kondrashova MN (2006) Nonezymatic formation of succinate in mitochondria under oxidative stress. Free Radic Biol Med 41: 56–64. 16781453 10.1016/j.freeradbiomed.2006.02.012

[9] Wu JL, Wu QP, Yang XF, Wei MK, Zhang JM, Huang Q, et al (2008) L-malate reverses oxidative stress and antioxidative defenses in liver and heart of aged rats. Physiol Res 57: 261–268. 17298203 10.33549/physiolres.931161

[10] Mittler R (2002) Oxidative stress, antioxidants and stress tolerance. Trends Plant Sci 7: 405–410. 12234732 10.1016/s1360-1385(02)02312-9

[11] Singh R, Lemire J, Mailloux RJ, Appanna VD (2008) A novel strategy involved in [corrected] anti-oxidative defense: the conversion of NADH into NADPH by a metabolic network. PLoS One 3: e2682 10.1371/journal.pone.0002682 18628998 PMC2443280

[12] Tie S, Bin R, Hong Z, Ximing Z, Yin Y, Han W, et al (2013) Metabolic flux ratio analysis and multi-objective optimization revealed a globally conserved and coordinated metabolic response of *E*. *coli* to paraquat-induced oxidative stress. Mol Biosyst 9: 121–132. 10.1039/c2mb25285f 23128557

[13] Chemler JA, Fowler ZL, McHugh KP, Koffas MA. (2010) Improving NADPH availability for natural product biosynthesis in *Escherichia coli* by metabolic engineering. Metab Eng 12(2): 96–104. 10.1016/j.ymben.2009.07.003 19628048

[14] Siedler S, Bringer S, Bott M (2011) Increased NADPH availability in *Escherichia coli*: improvement of the product per glucose ratio in reductive whole-cell biotransformation. Appl Microbiol Biotechnol 92(5): 929–37. 10.1007/s00253-011-3374-4 21670981

[15] Martinez I, Zhu J, Lin H, Bennett GN, San KY (2008) Replacing Escherichia coli NAD-dependent glyceraldehyde 3-phosphate dehydrogenase (GAPDH) with a NADP-dependent enzyme from *Clostridium acetobutylicum* facilitates NADPH dependent pathways. Metab Eng 10(6): 352–9. 10.1016/j.ymben.2008.09.001 18852061

[16] Jiang LY, Zhang YY, Li Z, Liu JZ (2013) Metabolic engineering of *Corynebacterium glutamicum* for increasing the production of L-ornithine by increasing NADPH availability. J Ind Microbiol Biotechnol 40(10): 1143–51. 10.1007/s10295-013-1306-2 23836141

[17] Papin JA, Price ND, Wiback SJ, Fell DA, Palsson BO (2003) Metabolic pathways in the post-genome era. Trends Biochem Sci 28: 250–258. 12765837 10.1016/S0968-0004(03)00064-1

[18] Steuer R, Gross T, Selbi J, Blasius B (2006) Structural kinetic modeling of metabolic networks. Proc Natl Acad Sci USA 103: 11868–11873. 16880395 10.1073/pnas.0600013103 PMC1524928

[19] Klamt S, Stelling J (2003) Two approaches for metabolic pathway analysis? Trends Biotechnol 21: 64–69. 12573854 10.1016/s0167-7799(02)00034-3

[20] Schuster S, Dandekar T, Fell DA (1999) Detection of elementary flux modes in biochemical networks: a promising tool for pathway analysis and metabolic engineering. Trends Biotechnol 17: 53–60. 10087604 10.1016/s0167-7799(98)01290-6

[21] Wiback SJ, Palsson BO (2002) Extreme pathway analysis of human red blood cell metabolism. Biophys J 83: 808–818. 12124266 10.1016/S0006-3495(02)75210-7 PMC1302188

[22] Stelling J, Klamt S, Bettenbrock K, Schuster S, Gilles ED (2002) Metabolic network structure determines key aspects of functionality and regulation. Nature 420: 190–193. 12432396 10.1038/nature01166

[23] de Figueiredo LF, Gossmann TI, Ziegler M, Schuster S (2011) Pathway analysis of NAD+ metabolism. Biochem J 439: 341–348. 10.1042/BJ20110320 21729004

[24] Jol SJ, Kummel A, Terzer M, Stelling J, Heinemann M (2012) System-level insights into yeast metabolism by thermodynamic analysis of elementary flux modes. PLoS Comput Biol 8: e1002415 10.1371/journal.pcbi.1002415 22416224 PMC3296127

[25] Schellenberger J, Park JO, Conrad TM, Palsson BO (2010) BiGG: a Biochemical Genetic and Genomic knowledgebase of large scale metabolic reconstructions. BMC Bioinformatics 11: 213 10.1186/1471-2105-11-213 20426874 PMC2874806

[26] Chang A, Scheer M, Grote A, Schomburg I, Schomburg D (2009) BRENDA, AMENDA and FRENDA the enzyme information system: new content and tools in 2009. Nucleic Acids Res 37: D588–592. 10.1093/nar/gkn820 18984617 PMC2686525

[27] Schwarz R, Musch P, von Kamp A, Engels B, Schirmer H, Schuster S et al (2005) YANA—a software tool for analyzing flux modes, gene-expression and enzyme activities. BMC Bioinformatics 6: 135 15929789 10.1186/1471-2105-6-135 PMC1175843

[28] von Kamp A, Schuster S (2006) Metatool 5.0: fast and flexible elementary modes analysis. Bioinformatics 22: 1930–1931. 16731697 10.1093/bioinformatics/btl267

[29] Supek F, Vlahovicek K (2004) INCA: synonymous codon usage analysis and clustering by means of self-organizing map. Bioinformatics 20: 2329–2330. 15059815 10.1093/bioinformatics/bth238

[30] Yeung KY, Haynor DR, Ruzzo WL (2001) Validating clustering for gene expression data. Bioinformatics 17: 309–318. 11301299 10.1093/bioinformatics/17.4.309

[31] Henry CS, Jankowski MD, Broadbelt LJ, Hatzimanikatis V (2006) Genome-scale thermodynamic analysis of *Escherichia coli* metabolism. Biophys J 90: 1453–1461. 16299075 10.1529/biophysj.105.071720 PMC1367295

[32] Henry CS, Broadbelt LJ, Hatzimanikatis V (2007) Thermodynamics-based metabolic flux analysis. Biophys J 92: 1792–1805. 17172310 10.1529/biophysj.106.093138 PMC1796839

[33] Bennett BD, Kimball EH, Gao M, Osterhout R, Van Dien SJ, Rabinowitz JD (2009) Absolute metabolite concentrations and implied enzyme active site occupancy in *Escherichia coli* . Nat Chem Biol 5: 593–599. 10.1038/nchembio.186 19561621 PMC2754216

[34] Chassagnole C, Noisommit-Rizzi N, Schmid JW, Mauch K, Reuss M (2002) Dynamic modeling of the central carbon metabolism of *Escherichia coli* . Biotechnol Bioeng 79: 53–73. 17590932 10.1002/bit.10288

[35] Jozefczuk S, Klie S, Catchpole G, Szymanski J, Cuadros-Inostroza A, Steinhauser D, et al (2010) Metabolomic and transcriptomic stress response of *Escherichia coli* . Mol Syst Biol 6: 364 10.1038/msb.2010.18 20461071 PMC2890322

[36] Jankowski MD, Henry CS, Broadbelt LJ, Hatzimanikatis V (2008) Group contribution method for thermodynamic analysis of complex metabolic networks. Biophys J 95: 1487–1499. 10.1529/biophysj.107.124784 18645197 PMC2479599

[37] Mavrovouniotis ML (1991) Estimation of standard Gibbs energy changes of biotransformations. J Biol Chem 266: 14440–14445. 1860851

[38] Siedler S, Bringer S, Blank LM, Bott M (2012) Engineering yield and rate of reductive biotransformation in *Escherichia coli* by partial cyclization of the pentose phosphate pathway and PTS-independent glucose transport. Appl Microbiol Biotechnol 93(4): 1459–67. 10.1007/s00253-011-3626-3 22002070 PMC3275745

[39] Siedler S, Lindner SN, Bringer S, Wendisch VF, Bott M (2013) Reductive whole-cell biotransformation with *Corynebacterium glutamicum*: improvement of NADPH generation from glucose by a cyclized pentose phosphate pathway using pfkA and gapA deletion mutants. Appl Microbiol Biotechnol 97(1): 143–52. 10.1007/s00253-012-4314-7 22851018 PMC3536970

[40] Ralser M, Wamelink MM, Latkolik S, Jansen EE, Lehrach H, Jakobs C (2009) Metabolic reconfiguration precedes transcriptional regulation in the antioxidant response. Nat Biotechnol 27: 604–605. 10.1038/nbt0709-604 19587661

[41] Berger A, Dohnt K, Tielen P, Jahn D, Becker J, Wittmann C (2014) Robustness and plasticity of metabolic pathway flux among uropathogenic isolates of *Pseudomonas aeruginosa* . PLoS One 9: e88368 10.1371/journal.pone.0088368 24709961 PMC3977821

[42] Stina K L, Sebastian N, Håvard S, Katharina N, Per B (2015) Fluxome study of *Pseudomonas fluorescens* reveals major reorganisation of carbon flux through central metabolic pathways in response to inactivation of the anti-sigma factor MucA. BMC Syst Biol 9: 6 10.1186/s12918-015-0148-0 25889900 PMC4351692

[43] Hanke T, Noh K, Noack S, Polen T, Bringer S, Sahm H, et al (2013) Combined fluxomics and transcriptomics analysis of glucose catabolism via a partially cyclic pentose phosphate pathway in *Gluconobacter oxydans* 621H. Appl Environ Microbiol 79(7): 2336–48. 10.1128/AEM.03414-12 23377928 PMC3623255

[44] Suga H, Matsuda F, Hasunuma T, Ishii J, Kondo A (2013) Implementation of a transhydrogenase-like shunt to counter redox imbalance during xylose fermentation in *Saccharomyces cerevisiae* . Appl Microbiol Biotechnol 97(4): 1669–78. 10.1007/s00253-012-4298-3 22851014

[45] Dghim AA, Dumont J, Hasenfratz-Sauder MP, Dizengremel P, Le Thiec D, Jolivet Y (2013) Capacity for NADPH regeneration in the leaves of two poplar genotypes differing in ozone sensitivity. Physiol Plant 148(1): 36–50. 10.1111/j.1399-3054.2012.01686.x 22978704

