# Supplementary material for: Elementary Flux Mode Analysis Revealed Cyclization Pathway as a Powerful Way for NADPH Regeneration of Central Carbon Metabolism
Source: PLoS One. 2015 Jun 18;10(6):e0129837. doi: 10.1371/journal.pone.0129837 (PMC4472234; doi:10.1371/journal.pone.0129837)
Supplement: S1 Appendix — (DOC) [file pone.0129837.s001.doc]

**S1 Appendix**

Abbreviation：

| 1 | ACALD | 'acetaldehyde dehydrogenase (acetylating)' | Acetaldehyde + Coenzyme-A + Nicotinamide-adenine-dinucleotide <==> Acetyl-CoA + H + Nicotinamide-adenine-dinucleotide-reduced |
| --- | --- | --- | --- |
| 2 | ACALDt | 'acetaldehyde reversible transport' | Acetaldehyde <==> Acetaldehyde |
| 3 | ACKr | 'acetate kinase' | Acetate + ATP <==> Acetyl-phosphate + ADP |
| 4 | ACONTa | 'aconitase (half-reaction A, Citrate hydro-lyase)' | Citrate <==> Cis-Aconitate + H_2_O |
| 5 | ACONTb | 'aconitase (half-reaction B, Isocitrate hydro-lyase)' | Cis-Aconitate + H_2_O <==> Isocitrate |
| 6 | ACt2r | 'acetate reversible transport via proton symport' | Acetate + H<==>Acetate + H |
| 7 | ADK1 | 'adenylate kinase' | AMP + ATP<==>ADP |
| 8 | AKGDH | '2-Oxoglutarate dehydrogenase' | 2-Oxoglutarate + Coenzyme-A + Nicotinamide-adenine-dinucleotide<==> CO_2_ + Nicotinamide-adenine-dinucleotide-reduced + Succinyl-CoA |
| 9 | AKGt2r | '2-oxoglutarate reversible transport via symport' | 2-Oxoglutarate + H<==>2-Oxoglutarate + H |
| 10 | ALCD2x | 'alcohol dehydrogenase (ethanol)' | Ethanol + Nicotinamide-adenine-dinucleotide<==>Acetaldehyde + H + Nicotinamide-adenine-dinucleotide-reduced |
| 11 | ATPM | 'ATP maintenance requirement' | ATP + H2O <==>ADP + H + Pi |
| 12 | ATPS4r | 'ATP synthase (four protons for one ATP)' | ADP + H + Pi<==>ATP + H_2_O + H |
| 13 | Biomass_ _core_w_GAM | 'Biomass Objective Function with GAM' | 3-Phospho-D-glycerate + Acetyl-CoA + ATP + D-Erythrose-4-phosphate + D-Fructose-6-phosphate + Glyceraldehyde-3-phosphate + D-Glucose-6-phosphate + L-Glutamine + L-Glutamate + H_2_O + Nicotinamide-adenine-dinucleotide + Nicotinamide-adenine-dinucleotide-phosphate-reduced + Oxaloacetate + Phosphoenolpyruvate + Pyruvate + D-Ribose-5-phosphate <==>ADP + 2-Oxoglutarate + Coenzyme-A + H + Nicotinamide-adenine-dinucleotide-reduced + Nicotinamide-adenine-dinucleotide-phosphate + Pi |
| 14 | CO_2_t | 'CO_2_ transporter via diffusion' | CO_2_ <==> CO_2_ |
| 15 | CS | 'citrate synthase' | Acetyl-CoA + H_2_O + Oxaloacetate<==>Citrate + Coenzyme-A + H |
| 16 | CYTBD | 'cytochrome oxidase bd (ubiquinol-8: 2 protons)' | H + O_2_ + Ubiquinol-8 <==> H_2_O + H + Ubiquinone-8 |
| 17 | D_LACt2 | 'D-lactate transport via proton symport' | H + Lactate<==>H + Lactate |
| 18 | ENO | 'enolase' | D-Glycerate-2-phosphate <==> H_2_O + Phosphoenolpyruvate |
| 19 | ETOHt2r | 'ethanol reversible transport via proton symport' | Ethanol + H<==>Ethanol + H |
| 20 | EX_ac_LPAREN_e_RPAREN_ | 'Acetate exchange' | Acetate |
| 21 | EX_acald_LPAREN_e_RPAREN_ | 'Acetaldehyde exchange' | Acetaldehyde |
| 22 | EX_akg_LPAREN_e_RPAREN_ | '2-Oxoglutarate exchange' | 2-Oxoglutarate |
| 23 | EX_co2_LPAREN_e_RPAREN_ | 'CO_2_ exchange' | CO_2_ |
| 24 | EX_etoh_LPAREN_e_RPAREN_ | 'Ethanol exchange' | Ethanol |
| 25 | EX_foLPAREN_e_RPAREN_ | 'Formate exchange' | Formate |
| 26 | EX_fru_LPAREN_e_RPAREN_ | 'D-Fructose exchange' | D-Fructose |
| 27 | EX_fum_LPAREN_e_RPAREN_ | 'Fumarate exchange' | Fumarate |
| 28 | EX_glc_LPAREN_e_RPAREN_ | 'D-Glucose exchange' | D-Glucose |
| 29 | EX_gln_L_LPAREN_e_RPAREN_ | 'L-Glutamine exchange' | L-Glutamine |
| 30 | EX_glu_L_LPAREN_e_RPAREN_ | 'L-Glutamate exchange' | L-Glutamate |
| 31 | EX_h_LPAREN_e_RPAREN_ | 'H+ exchange' | H |
| 32 | EX_h_2_o_LPAREN_e_RPAREN_ | 'H_2_O exchange' | H_2_O |
| 33 | EX_lac_D_LPAREN_e_RPAREN_ | 'D-Lactate exchange' | Lactate |
| 34 | EX_mal_L_LPAREN_e_RPAREN_ | 'L-Malate exchange' | L-Malate |
| 35 | EX_nh4_LPAREN_e_RPAREN_ | 'Ammonium exchange' | Ammonium |
| 36 | EX_o2_LPAREN_e_RPAREN_ | 'O_2_ exchange' | O_2_ |
| 37 | EX_pi_LPAREN_e_RPAREN_ | 'Phosphate exchange' | Pi |
| 38 | EX_pyLPAREN_e_RPAREN_ | 'Pyruvate exchange' | Pyruvate |
| 39 | EX_succ_LPAREN_e_RPAREN_ | 'Succinate exchange' | Sucinnate |
| 40 | FBA | 'fructose-bisphosphate aldolase' | D-Fructose-1-6-bisphosphate<==>Dihydroxyacetone-phosphate + Glyceraldehyde-3-phosphate |
| 41 | FBP | 'fructose-bisphosphatase' | D-Fructose-1-6-bisphosphate + H_2_O <==> D-Fructose-6-phosphate + Pi |
| 42 | FORt2 | 'formate transport via proton symport (uptake only)' | Formate + H<==>Formate + H |
| 43 | FORti | 'formate transport via diffusion' | Formate<==>Formate |
| 44 | FRD7 | 'fumarate reductase' | Fumarate + Ubiquinol-8 <==> Ubiquinone-8 + Succinate |
| 45 | FRUpts2 | 'Fructose transport via PEP:Pyr PTS (f6p generating)' | D-Fructose + Phosphoenolpyruvate<==> D-Fructose-6-phosphate + Pyruvate |
| 46 | FUM | 'fumarase' | Fumarate + H2O <==>Malate |
| 47 | FUMt2_2 | 'Fumarate transport via proton symport (2 H)' | Fumarate + H<==>Fumarate + H |
| 48 | G6PDH2r | 'glucose 6-phosphate dehydrogenase' | D-Glucose-6-phosphate + Nicotinamide-adenine-dinucleotide-phosphate<==> 6-phospho-D-glucono-1-5-lactone + H + Nicotinamide-adenine-dinucleotide-phosphate-reduced |
| 49 | GAPD | 'glyceraldehyde-3-phosphate dehydrogenase' | Glyceraldehyde-3-phosphate + Nicotinamide-adenine-dinucleotide + Pi<==> 3-Phospho-D-glyceroyl-phosphate + H + Nicotinamide-adenine-dinucleotide-reduced |
| 50 | GLCpts | 'D-glucose transport via PEP:Pyr PTS' | D-Glucose + Phosphoenolpyruvate<==> D-Glucose-6-phosphate + Pyruvate |
| 51 | GLNS | 'glutamine synthetase' | ATP + L-Glutamate + Ammonia <==>ADP + L-Glutamine + H + Pi |
| 52 | GLNabc | 'L-glutamine transport via ABC system' | ATP + L-Glutamine + H_2_O <==>ADP + L-Glutamine + H + Pi |
| 53 | GLUDy | 'glutamate dehydrogenase (NADP)' | L-Glutamate + H_2_O + Nicotinamide-adenine-dinucleotide-phosphate<==>2-Oxoglutarate + H + Nicotinamide-adenine-dinucleotide-phosphate-reduced + Ammonia |
| 54 | GLUN | 'glutaminase' | L-Glutamine + H_2_O <==>L-Glutamate + Ammonia |
| 55 | GLUSy | 'glutamate synthase (NADPH)' | 2-Oxoglutarate + L-Glutamine + H + Nicotinamide-adenine-dinucleotide-phosphate-reduced<==>L-Glutamate + Nicotinamide-adenine-dinucleotide-phosphate |
| 56 | GLUt2r | 'L-glutamate transport via proton symport, reversible (periplasm)' | L-Glutamate + H<==>L-Glutamate + H |
| 57 | GND | 'phosphogluconate dehydrogenase' | 6-Phospho-D-gluconate + Nicotinamide-adenine-dinucleotide-phosphate<==> CO_2_ + Nicotinamide-adenine-dinucleotide-phosphate-reduced + D-Ribulose-5-phosphate |
| 58 | H_2_Ot | 'H_2_O transport via diffusion' | H_2_O <==> H_2_O |
| 59 | ICDHyr | 'isocitrate dehydrogenase (NADP)' | Isocitrate + Nicotinamide-adenine-dinucleotide-phosphate<==>2-Oxoglutarate + CO_2_ + Nicotinamide-adenine-dinucleotide-phosphate-reduced |
| 60 | ICL | 'Isocitrate lyase' | Isocitrate<==>Glyoxylate + Succinate |
| 61 | LDH_D | 'D lactate dehydrogenase' | Lactate + Nicotinamide-adenine-dinucleotide<==>H + Nicotinamide-adenine-dinucleotide-reduced + Pyruvate |
| 62 | MALS | 'malate synthase' | Acetyl-CoA + Glyoxylate + H_2_O <==>Coenzyme-A + H + Malate |
| 63 | MALt2_2 | 'Malate transport via proton symport (2 H)' | H + L-Malate<==>H + Malate |
| 64 | MDH | 'malate dehydrogenase' | Malate + Nicotinamide-adenine-dinucleotide<==>H + Nicotinamide-adenine-dinucleotide-reduced + Oxaloacetate |
| 65 | ME(NAD) | 'malic enzyme (NAD)' | Malate + Nicotinamide-adenine-dinucleotide<==> CO_2_ + Nicotinamide-adenine-dinucleotide-reduced + Pyruvate |
| 66 | ME(NADP) | 'malic enzyme (NADP)' | Malate + Nicotinamide-adenine-dinucleotide-phosphate<==> CO_2_ + Nicotinamide-adenine-dinucleotide-phosphate-reduced + Pyruvate |
| 67 | NADH16 | 'NADH dehydrogenase (ubiquinone-8 & 3 protons)' | H + Nicotinamide-adenine-dinucleotide-reduced + Ubiquinone-8 <==>H + Nicotinamide-adenine-dinucleotide + Ubiquinol-8 |
| 68 | NADTRHD | 'NAD transhydrogenase' | Nicotinamide-adenine-dinucleotide + Nicotinamide-adenine-dinucleotide-phosphate-reduced<==>Nicotinamide-adenine-dinucleotide-reduced + Nicotinamide-adenine-dinucleotide-phosphate |
| 69 | NH_4_t | 'ammonia reversible transport' | Ammonium <==> Ammonia |
| 70 | O_2_t | O_2_ transport via diffusion' | O_2_ <==> O_2_ |
| 71 | PDH | 'pyruvate dehydrogenase' | Coenzyme-A + Nicotinamide-adenine-dinucleotide + Pyruvate<==>Acetyl-CoA + CO_2_ + Nicotinamide-adenine-dinucleotide-reduced |
| 72 | PFK | 'phosphofructokinase' | ATP + D-Fructose-6-phosphate <==>ADP + D-Fructose-1-6-bisphosphate + H |
| 73 | PFL | 'pyruvate formate lyase' | Coenzyme-A + Pyruvate <==>Acetyl-CoA + Formate |
| 74 | PGI | 'glucose-6-phosphate isomerase' | D-Glucose-6-phosphate <==> D-Fructose-6-phosphate |
| 75 | PGK | 'phosphoglycerate kinase' | 3-Phospho-D-glycerate + ATP <==> 3-Phospho-D-glyceroyl-phosphate + ADP |
| 76 | PGL | '6-phosphogluconolactonase' | 6-phospho-D-glucono-1-5-lactone + H_2_O <==> 6-Phospho-D-gluconate + H |
| 77 | PGM | 'phosphoglycerate mutase' | D-Glycerate-2-phosphate <==> 3-Phospho-D-glycerate |
| 78 | PIt2r | 'phosphate reversible transport via proton symport' | H + Pi<==>H + Pi |
| 79 | PPC | 'phosphoenolpyruvate carboxylase' | CO_2_ + H_2_O + Phosphoenolpyruvate<==>H + Oxaloacetate + Pi |
| 80 | PPCK | 'phosphoenolpyruvate carboxykinase' | ATP + Oxaloacetate<==>ADP + CO_2_ + Phosphoenolpyruvate |
| 81 | PPS | 'phosphoenolpyruvate synthase' | ATP + H_2_O + Pyruvate<==>AMP + H + Phosphoenolpyruvate + Pi |
| 82 | PTAr | 'phosphotransacetylase' | Acetyl-CoA + Pi<==>Acetyl-phosphate + Coenzyme-A |
| 83 | PYK | 'pyruvate kinase' | ADP + H + Phosphoenolpyruvate<==>ATP + Pyruvate |
| 84 | PYRt2r | 'pyruvate reversible transport via proton symport' | H + Pyruvate<==>H + Pyruvate |
| 85 | RPE | 'ribulose 5-phosphate 3-epimerase' | D-Ribulose-5-phosphate <==> D-Xylulose-5-phosphate |
| 86 | RPI | 'ribose-5-phosphate isomerase' | D-Ribose-5-phosphate <==> D-Ribulose-5-phosphate |
| 87 | SUCCt2_2 | 'succinate transport via proton symport (2 H)' | H + Sucinnate<==>H + Succinate |
| 88 | SUCCt3 | 'succinate transport out via proton antiport' | H + Succinate<==>H + Sucinnate |
| 89 | SUCDi | 'succinate dehydrogenase (irreversible)' | Ubiquinone-8 + Succinate<==>Fumarate + Ubiquinol-8 |
| 90 | SUCOAS | 'succinyl-CoA synthetase (ADP-forming)' | ATP + Coenzyme-A + Succinate<==>ADP + Pi + Succinyl-CoA |
| 91 | TALA | 'transaldolase' | Glyceraldehyde-3-phosphate + Sedoheptulose-7-phosphate <==> D-Erythrose-4-phosphate + D-Fructose-6-phosphate |
| 92 | THD2 | 'NAD(P) transhydrogenase' | H + Nicotinamide-adenine-dinucleotide-reduced + Nicotinamide-adenine-dinucleotide-phosphate<==>H + Nicotinamide-adenine-dinucleotide + Nicotinamide-adenine-dinucleotide-phosphate-reduced |
| 93 | TKT1 | 'transketolase' | D-Ribose-5-phosphate + D-Xylulose-5-phosphate <==> Glyceraldehyde-3-phosphate + Sedoheptulose-7-phosphate |
| 94 | TKT2 | 'transketolase' | D-Erythrose-4-phosphate + D-Xylulose-5-phosphate <==> D-Fructose-6-phosphate + Glyceraldehyde-3-phosphate |
| 95 | TPI | 'triose-phosphate isomerase' | Dihydroxyacetone-phosphate<==> Glyceraldehyde-3-phosphate |
| 96 | EDD1 | ‘6-phosphogluconate dehydratase’ | 6-Phospho-D-gluconate <==> 2-keto-3-deoxyphosphogluconate |
| 97 | EDD2 | ‘2-keto-3-deoxyphosphogluconate aldolase’ | 2-keto-3-deoxyphosphogluconate <==> Glyceraldehyde-3-phosphate + Pyruvate |
| 98 | TOTAL_NADPH |  | V_G6PDH2r_ + V_GLUDy_ –V_GLUSy_ + V_GND_ + V_ICDHyr_ + V_ME2_ |
