# Supplementary material for: Elementary Flux Mode Analysis Revealed Cyclization Pathway as a Powerful Way for NADPH Regeneration of Central Carbon Metabolism
Source: PLoS One. 2015 Jun 18;10(6):e0129837. doi: 10.1371/journal.pone.0129837 (PMC4472234; doi:10.1371/journal.pone.0129837)
Supplement: S2 Appendix — (DOC) [file pone.0129837.s002.doc]

**S2 Appendix**: Nomenclature

AcCoA: acetyl-coenzyme A

AKG: ɑ-Ketoglutarate

E4P: erythrose-4-phosphate

F6P: fructose-6-phosphate

GLC: glucose

G6P: glucose-6-phosphate

GAP: glyceraldehyde-3-phosphate

OAA: oxaloacetic acid

SUC: succinate

GLY: glycine

Glyoxy: glyoxylate

ICIT: isocitrate

MAL: malate

PYR: pyruvate

PEP: phosphoenolpyruvate

PGA: 3-phosphoglycerate

R5P: ribose 5-phosphate

Ru5P: ribulose 5-phosphate

S7P: sedoheptulose 7-phosphate

X5P: xylulose 5-phosphate

Ery4P: erythrose 4-phosphate

SER: serine

T3P: triose-3-phosphate

DHAP: dihydroxyacetone-phosphate

3PG: 3-Phospho-D-glycerate

2PG: D-Glycerate-2-phosphate

Ube-8: ubiquinone-8

Ubl-8: ubiquinol-8
